# Loss of genetic diversity as a signature of apricot domestication and diffusion into the Mediterranean Basin

**DOI:** 10.1186/1471-2229-12-49

**Published:** 2012-04-17

**Authors:** Hedia Bourguiba, Jean-Marc Audergon, Lamia Krichen, Neila Trifi-Farah, Ali Mamouni, Samia Trabelsi, Claudio D’Onofrio, Bayram M Asma, Sylvain Santoni, Bouchaib Khadari

**Affiliations:** 1 INRA, UMR 1334 Amélioration Génétique et Adaptation des Plantes (AGAP), F-34398, Montpellier, France; 2 INRA Centre PACA – UR1052 GAFL, Domaine St Maurice, BP94, 84143, Montfavet Cedex, France; 3 Faculté des Sciences de Tunis, Laboratoire de Génétique Moléculaire, Immunologie et Biotechnologie, Campus Universitaire, 2092, El Manar, Tunisia; 4 INRA, UR Amélioration des Plantes et Conservation des Ressources Phytogénétiques, Meknès, Morocco; 5 Université de Blida, Chaire d’arboriculture, Blida, Algeria; 6 Department of Fruit Science and Plant Protection of Woody Species “G. Scaramuzzi”, section of Fruit Science, University of Pisa, Via del Borghetto, 80, 56124, Pisa, Italy; 7 Department of Biology, Inonu University, Malatya, 44280, Turkey; 8 CBNMED, UMR 1334 AGAP, F-34398, Montpellier, France

## Abstract

**Background:**

Domestication generally implies a loss of diversity in crop species relative to their wild ancestors because of genetic drift through bottleneck effects. Compared to native Mediterranean fruit species like olive and grape, the loss of genetic diversity is expected to be more substantial for fruit species introduced into Mediterranean areas such as apricot (*Prunus armeniaca* L.), which was probably primarily domesticated in China. By comparing genetic diversity among regional apricot gene pools in several Mediterranean areas, we investigated the loss of genetic diversity associated with apricot selection and diffusion into the Mediterranean Basin.

**Results:**

According to the geographic origin of apricots and using Bayesian clustering of genotypes, Mediterranean apricot (207 genotypes) was structured into three main gene pools: ‘Irano-Caucasian’, ‘North Mediterranean Basin’ and ‘South Mediterranean Basin’. Among the 25 microsatellite markers used, only one displayed deviations from the frequencies expected under neutrality. Similar genetic diversity parameters were obtained within each of the three main clusters using both all SSR loci and only 24 SSR loci based on the assumption of neutrality. A significant loss of genetic diversity, as assessed by the allelic richness and private allelic richness, was revealed from the ‘Irano-Caucasian’ gene pool, considered as a secondary centre of diversification, to the northern and southwestern Mediterranean Basin. A substantial proportion of shared alleles was specifically detected when comparing gene pools from the ‘North Mediterranean Basin’ and ‘South Mediterranean Basin’ to the secondary centre of diversification.

**Conclusions:**

A marked domestication bottleneck was detected with microsatellite markers in the Mediterranean apricot material, depicting a global image of two diffusion routes from the ‘Irano-Caucasian’ gene pool: North Mediterranean and Southwest Mediterranean. This study generated genetic insight that will be useful for management of Mediterranean apricot germplasm as well as genetic selection programs related to adaptive traits.

## Background

Domestication of plants is a complex evolutionary process in which human selection favours phenotypic transitions making them more useful for humans and better adapted to landscape management. It is a crucial step in the evolution of crop species since humans have an important impact on their origins and changes. Moreover, selection pressure and local diversification lead to an ongoing process [1]. Two major impacts on plant diversity result from domestication. Firstly, changes in traits selected for human use, called the “domestication syndrome” [2], lead to selection signatures at specific loci [3,4]. In fact, according to the intensity of the selection process, the degree of change in populations can vary along a continuum from their wild ancestors to the domesticated populations, which cannot reproduce or survive without human intervention. Several highly domesticated plants such as maize, rice and wheat express domestication traits and have lost their ability to survive on their own in the wild [5]. Other crops like trees and forage are generally considered to be partially domesticated while conserving some ability to survive in natural environments [6]. In seed-propagated crops, domesticated types are characterized by a lack of seed dispersal at maturity and a lack of seed dormancy [7], while in clonally propagated crops, the reduction of sexual fertility and adaptations facilitating vegetative propagation have generally been reported [8].

The second major consequence of domestication is the reduction of genetic diversity in crops relative to their wild progenitors due to human selection and genetic drift through bottleneck effects [9]. Contrary to selection which only affects genetic diversity at target genes [3,4], bottleneck processes reduce neutral genetic diversity across the entire genome [10-12]. The strength of genetic drift during the domestication bottleneck is determined by its duration and the effective population size [13]. Thus, according to their life-history traits and evolutionary history, diversity loss differs considerably among crop plants. The reduction of gene diversity in crops compared to wild relatives has been observed in soybean (34%), maize (38%) and wheat (70–90%) [10-12]. However, introgressive hybridization between domesticated forms and their wild relatives has often expanded genetic diversity, counteracting the effects of the initial domestication bottleneck [14].

For perennial fruit species, domestication means changing the reproductive biology from sexual reproduction (in the wild) to vegetative propagation (under cultivation) [15]. Few studies have reported the impact of the domestication history and how bottleneck effects may reduce the genetic diversity of crops relative to the wild relatives. Miller and Schall [16] provided phylogeographic evidence of multiple domestication of a cultivated fruit tree, *Spondias purpurea*, within the Mesoamerican centre of domestication. About 29% of the total diversity was not recovered in wild populations, suggesting that either new alleles have arisen during cultivation or, alternatively, contemporary extinction of tropical dry forests has occurred in Mesoamerican areas leading to genetic erosion of the wild gene pool. In Mediterranean zones, only a weak bottleneck effect on diversity in olive and grapevine was observed when comparing the wild and cultivated forms [17,18]. For *Prunus* species, Mariette et al. [19] reported that in the case of sweet cherry a marked genetic bottleneck due to plant breeding was detected at microsatellite loci (40%) and at the *S*-locus coding for a gametophytic self-incompatibility (GSI) system (30%). However, the domestication bottleneck, as estimated by the loss of genetic diversity between wild cherry and landraces, was not detected by SSR markers but only observed at the *S*-locus (20%).

Apricot*, Prunus armeniaca* L., is a stone-fruit species that is grown commercially worldwide in all temperate regions. The numerous cultivars are highly adapted to restricted areas [20]. Apricot is clonally propagated through grafting but it is also seed-propagated, mainly in oasis agroecosystems. The Mediterranean area accounts for over 50% of the worldwide production [21]. Apricot was probably initially domesticated in China where wild apricot is found [22]. Following several collection expeditions through the major agricultural areas of the world and based on morphological data, Vavilov [23] proposed an explanation to determine the centre of origin of cultivated plants and described three regions as centres of origin for apricot: a Chinese centre, a Central Asian centre and a Near East centre. The latter centre included apricot from the Irano-Caucasian area (Iran, Caucasia and Turkey) and was considered as a secondary centre of cultivar diversification because of its presumed intermediate geographic position between the main area of cultivation of the domesticate and the distribution of the wild species [24]. On the basis of morphological characters and pomological descriptions, four major eco-geographical apricot groups were defined [25]: (*i*) The Central Asian group is the oldest and most diversified. The cultivars are self-incompatible apricots and have high-frost requirements; (*ii*) the Dzhungar-Zailij group includes self-incompatible small-fruited cultivars; (*iii*) the Irano-Caucasian group mostly encompasses self-incompatible apricots with reduced chilling requirements; and (*iv*) the European group is the most recent one, including self-compatible cultivars. According to the morphological characters, the expansion of apricot species into the Mediterranean Basin may have occurred in two waves along two distinct major apricot diffusion routes [24,26]: the first one being brought by the Arabs through the Near East and North Africa, and the second through Hungary and Central Europe. However, Kostina [25] reported only one major route from the Irano-Caucasian area to the Mediterranean Basin. Hence, apricot domestication and its diffusion into the Mediterranean Basin are still debated issues.

Recently, improvements in neutral molecular markers have significantly increased the capacity of genetic characterization and relationship studies in different apricot cultivars, generating important information relating to the genetic variability, selection processes and breeding history of this crop at a large spatial scale. In this setting, using both microsatellite and AFLP markers, apricot accessions collected from different eco-geographical groups (Europe, North America, Irano-Caucasia, Central Asia) have been grouped according to their geographical origin and pedigree information supporting the history of apricot diffusion from its centre of origin [27-31]. However, these studies considered material collections, including both traditional cultivars and selected accessions derived from breeding programs, and did not take the difference between vegetative and seed propagation into account.

In Europe, clonal propagation through grafting and cuttings was used for a long time. Nevertheless, much of the remaining variability was from a mix between seed and grafting propagated apricots (i.e. Vesuvian apricots, Roussillon apricots, Peloponnese apricots) [32]. In North Africa ( Algeria, Morocco, and Tunisia), apricot germplasm contained accessions propagated by grafting, but also by seeds specifically located in oasian regions. A fine-scale genetic diversity study conducted using AFLP markers at the within-population level, focusing on Tunisian grafted apricot cultivars, supported the assumption of few introduced genotypes that have been firstly propagated by seeds [33]. Analysing a larger set of Tunisian apricots, including both vegetatively propagated cultivars and seed propagated accessions, Bourguiba et al. [34] identified two main gene pools according to their propagation mode and confirmed the assumption that these two gene pools shared the same origin.

A gradient of decreasing genetic diversity from east to west was proposed by Hagen et al. [35] among the four identified apricot groups: ‘Diversification’, ‘Adaptive Diversity’, ‘Continental Europe’ and ‘Mediterranean Basin’, which could be related to the apricot diffusion process. However, this study was limited to a small sample (only 50 accessions) and based on a phenetic approach related to the geographic origin and the phenotypic characters of the cultivars.

We still have incomplete knowledge of apricot domestication and diffusion into the Mediterranean Basin based on the assumption of a genetic diversity decrease [35] and balancing between one [25] or two major diffusion routes [24,26]. This was the first study generating insight into these evolutionary and historical processes following a genetic structure analysis in apricot which included a large sample size of local Mediterranean material and involved microsatellite markers as well as a model-based Bayesian clustering approach. Owing to their transferability across *Prunus* species, simple sequence repeat (SSR) markers have been widely used in variability studies and linkage map construction [36]. A set of 25 microsatellite markers was selected according to their polymorphism in apricot cultivars and their mapping over the *Prunus* genome.

The goal of this study was to clarify the history of the apricot domestication process in the Mediterranean area through an analysis of genotypes originating from Algeria, France, Iran, Italy, Morocco, Spain, Tunisia and Turkey. We specifically addressed the following questions: (*i*) What is the genetic structure of Mediterranean apricots compared to Irano-Caucasian germplasm? (*ii*) Is there a loss of genetic diversity from the Near-Eastern secondary centre of diversification to the extreme south-western Mediterranean area? and (*iii*) Can distinct apricot diffusion routes be identified throughout the Mediterranean Basin?

## Results

### SSR polymorphism

A total of 257 alleles was detected across the 25 SSR loci used, ranging from 5 (BPPCT001) to 18 per locus (UDP98-409; Table 1). The average number of alleles per locus was 10.28 but dropped to 3.96 when rare alleles were removed (i.e. with a frequency of less than 5%). The number of alleles per locus with a frequency higher than 5% ranged from 1 (UDP96-018) to 8 (UDP98-409). The average PIC value for the 25 loci was 0.586 and the most informative locus was UDP98-409 (0.861). Only three among the 25 SSR loci displayed a significant heterozygosity deficit (*P* < 0.01; BPPCT004, Ma040a and UDP97-402 loci; see Additional file 1: Table S1) in apricot from the Irano-Caucasian area (Iran and Turkey), which is considered as a secondary diversification zone [24], indicating the lack of null alleles even though most of SSR loci were not specifically developed in apricot [37-42].

**Table 1:** Genetic diversity scored at 25 mapped loci in the 207 apricot accessions

**Locus**	**LG**	***N***_***A***_	***N***_***A,P***_	**PIC**	***H***_***e***_	***H***_***o***_
AMPA109^β^	1	8	2	0.268	0.279	0.217
CPPCT034^¤^	1	9	2	0.425	0.478	0.454
UDP96-018^‡^	1	6	1	0.099	0.102	0.087
AMPA116^β^	2	11	6	0.766	0.795	0.686
BPPCT001^£^	2	5	2	0.365	0.465	0.468
BPPCT004^£^	2	12	5	0.780	0.806	0.652
BPPCT030^£^	2	8	5	0.745	0.783	0.705
AMPA101^β^	3	8	4	0.618	0.662	0.584
AMPA119^β^	3	8	2	0.316	0.340	0.280
BPPCT040^£^	4	10	2	0.417	0.496	0.415
UDP97-402^‡^	4	10	3	0.511	0.567	0.458
AMPA105^β^	5	12	6	0.741	0.772	0.690
BPPCT017^£^	5	10	3	0.514	0.569	0.560
BPPCT038^£^	5	13	5	0.746	0.781	0.657
AMPA100^β^	6	9	5	0.700	0.733	0.686
BPPCT008^£^	6	12	6	0.777	0.805	0.705
BPPCT025^£^	6	9	3	0.569	0.621	0.550
CPPCT030^¤^	6	16	6	0.739	0.764	0.710
Ma014a^#^	6	6	2	0.461	0.556	0.478
Ma040a^#^	6	11	3	0.573	0.622	0.487
UDP98-412^§^	6	11	4	0.643	0.681	0.555
CPPCT022^¤^	7	15	4	0.777	0.806	0.715
CPPCT033^¤^	7	9	4	0.443	0.464	0.396
CPPCT006^¤^	8	11	6	0.795	0.820	0.700
UDP98-409^‡^	8	18	8	0.861	0.876	0.739
Mean		10.28	3.96	0.586	0.626	0.545

LG linkage group position; *N*_*A *_number of alleles per locus; *N*_*A,P *_number of alleles with higher than 5% frequency; PIC polymorphic information content; *H*_*e *_expected heterozygosity; *H*_*o *_observed heterozygosity.

Primers developed by: ^β^Hagen et al. [37]; ^£^Dirlewanger et al. [38]; ^¤^Aranzana et al. [39]; ^#^Yamamoto et al. [40]; ^‡^Cipriani et al. [41]; ^§^Testolin et al. [42].

The Nei’s genetic diversity ranged from 0.102 (UDP96-018) to 0.876 (UDP98-409), with an average of 0.626 (Table 1), suggesting that the examined Mediterranean apricot germplasm enclosed higher polymorphism than reported in previous studies [27-30]. Compared to peach, apricot had higher polymorphism and was more diversified as confirmed by the number of alleles per locus and the observed heterozygosity, which was significantly higher on the 11 *SSR* loci that were used in both studies [43] (Mann–Whitney U-test, *P* < 0.05; the average number of alleles per locus was 11.80 in apricot and 7.64 in peach).

### Genetic structure and diversity within apricot geographic groups

**Figure 1:**
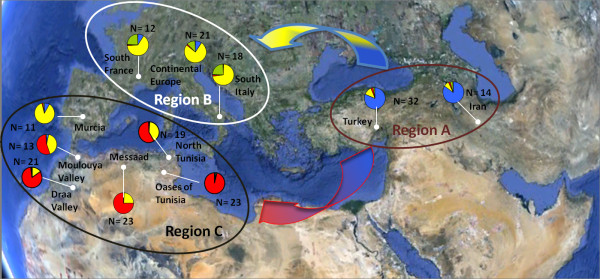
**Origin of the 207 apricot accessions classified into 11 apricot groups and three regions: A, B and C as defined according to their spatial and genetic proximity .** Algerian, Moroccan and Tunisian apricots were sampled in situ. The remaining accessions originated from an ex situ collection (see Additional file 2: Table S2). Region **A** = Iran and Turkey; Region **B** = Continental Europe, southern France and southern Italy; Region **C** = Murcia, northern Tunisia, Moulouya Valley, Messaad, Oases of Tunisia and Draa Valley. Colours correspond to genetic clusters defined by STRUCTURE analysis with cluster 1 in *blue*, cluster 2 in *green*, cluster 3 in *yellow* and cluster 4 in *red*.

According to the geographic origin of the studied apricot accessions, we defined eleven groups (Figure 1; see Additional file 2: Table S2). They displayed substantial genetic differentiation since the average *F*_*ST*_ value was 0.111, ranging from 0.024 for pairwise comparisons between the Iran and Turkey groups to 0.195 between the Murcia and Oases of Tunisia groups (see Additional file 3: Table S3). All pairwise *F*_*ST*_ values were significant at *P* < 10–6, except for the one observed between South Italy and Murcia, which was significant at *P* < 10–4.

**Figure 2:**
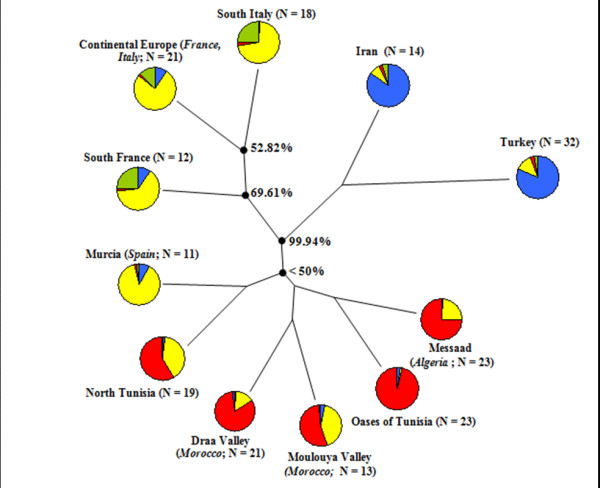
**Neighbor-joining clustering of geographic groups based on pairwise Nei’s genetic distance values, as well as the distribution of the genetic clusters within each of them. **Colours correspond to genetic clusters defined by the STRUCTURE analysis, as reported in Figure 3, with cluster 1 in blue, cluster 2 in green, cluster 3 in yellow and cluster 4 in red. Numbers next to nodes indicate bootstrap support percentages in 10000 pseudoreplicates.

Genetic relationships among the defined apricot groups were assessed based on Nei’s [44] genetic distances and the Neighbor-joining algorithm (Figure 2). According to the bootstrap values, the 11 apricot groups were classified into three regions (A, B and C; Figures 1 and 2). Region A, including Iran and Turkey groups (Iran-Caucasian region), was clearly distinguished from the remaining groups by a high bootstrap support of 99.94% (Figure 2). Region B, including Continental Europe, South France and South Italy groups, was defined by a bootstrap value of 69.61%. The third region (region C) included the five apricot groups from North Africa area as well as Murcia group, supported by a weak bootstrap value (35.81%; Figure 2). Based on the AMOVA analysis, the genetic variance was about 7% among these three defined regions and 14% among apricot groups per region (Table 2).

**Table 2:** Partitioning of variance within and among apricot groups and regions (average over 25 loci)

**Source of variation**	***df***	**Sum of squares**	**Variance components**	**Percentage of variation**
Among regions	2	311.75	1.353	7*
Among groups/regions	8	488.82	2.606	14*
Within groups	196	2840.47	14.492	79*
Total	206	3641.04	18.451	

The group level considered the eleven apricot geographic groups and the region level encompassed the three main geographic regions: A (Iran and Turkey), B (Continental Europe, southern France and southern Italy), and C (Murcia, northern Tunisia, Moulouya Valley, Messaad, Oases of Tunisia and Draa Valley).

*df *degrees of freedom.

* *P* < 0.001 based on 999 permutations.

The mean number of accessions per group was 18.81, ranging from 11 for the Murcia group to 32 for the Turkey group (Table 3). The levels of the genetic diversity estimators measured within these geographic groups differed: Iran and Turkey groups (region A), had the highest expected heterozygosity values, with 0.655 and 0.630, respectively; while the Oases of Tunisia and Draa Valley groups (region C) had the lowest ones, with 0.487 and 0.474, respectively. The observed heterozygosity was highest for South Italy (0.633) and lowest for Draa Valley (0.379). For the following groups: Turkey, Murcia, Moulouya Valley and Draa Valley, the F_*IS*_ values showed a significant heterozygosity deficit (Table 3).

**Table 3:** Genetic diversity within apricot geographic groups and regions

**Groups**	**Accessions number**	***H***_***o***_	***H***_***e***_	***F***_***IS***_	***N***_***A***_	***A***_***r***_	***A***_***pr***_
Iran	14	0.623	0.655	0.051	145	5.03^a^	0.47^a^
Turkey	32	0.585	0.630	0.073*	174	4.88^a^	0.43^a^
**Region A**	**46**	**0.596**	**0.645**	0.076**	**199**	**7.43**^**b**^	**1.86**^**b**^
Continental Europe	21	0.617	0.619	0.004	138	4.43^a^	0.35^a^
South France	12	0.623	0.609	−0.024	110	4.16^a^	0.09^a^
South Italy	18	0.633	0.579	−0.098	101	3.61^a^	0.07^a^
**Region B**	**51**	**0.624**	**0.623**	**−0.003**	**156**	**5.77**^**b**^	**0.79**^**b**^
Murcia	11	0.487	0.567	0.146***	95	3.73^a^	0.05^a^
North Tunisia	19	0.570	0.551	−0.036	100	3.48^a^	0.08^a^
Moulouya Valley	13	0.428	0.535	0.207*	96	3.52^a^	0.03^a^
Messaad	23	0.553	0.500	−0.108	97	3.36^a^	0.01^a^
Oases of Tunisia	23	0.489	0.487	−0.004	97	3.34^a^	0.02^a^
Draa Valley	21	0.379	0.474	0.204***	100	3.38^a^	0.01^a^
**Region C**	**110**	**0.488**	**0.569**	0.143***	**175**	**5.57**^**b**^	**0.80**^**b**^
*P *(Region A *vs.* B)		0.817	0.513		0.020^$^	0.017^$^	4.8 10^-5$$$^
*P *(Region A *vs.* C)		0.062	0.189		0.147	0.006^$$^	2.8 10^-5$$$^
*P *(Region B *vs.* C)		0.009^$$^	0.489		0.476	0.701	0.550

*H*_*o *_observed heterozygosity; *H*_*e *_expected heterozygosity; *F*_*IS *_fixation index values; Exact test significant at * *P* < 0.001, ** *P* < 0.0001, and *** *P* < 10^-5^.

*N*_*A *_total number of alleles per group; *A*_*r *_allelic richness; *A*_*pr *_private allelic richness.

Probability of independency between two regions using the two-tailed Mann-Whitney’s U test. ^$^*P* < 0.05; ^$$^*P* <0.01; ^$$$^*P* < 0.001.

^a ^standardized at maximum value G = 19 individuals per group; ^b^ standardized at maximum value G = 70 individuals per region.

As the number of alleles observed in a group is highly dependent on the sample size, the allelic richness and private allelic richness were computed for each group and region (Table 3). The highest allelic richness was detected for the Iran and Turkey groups (region A), with 5.03 and 4.88, respectively, while the lowest value was noted in the Draa Valley (3.38), Messaad (3.36) and Oases of Tunisia (3.34) groups belonging to region C. Similar results were obtained when computing the private allelic richness. Thus, region A had a significant upper level of genetic diversity in terms of allelic richness, private allelic richness and expected heterozygosity (Table 3), reflecting a decrease in genetic diversity from the eastern (region A) to the south-western (region C) Mediterranean Basin.

### Model-based Bayesian clustering analysis

**Figure 3:**
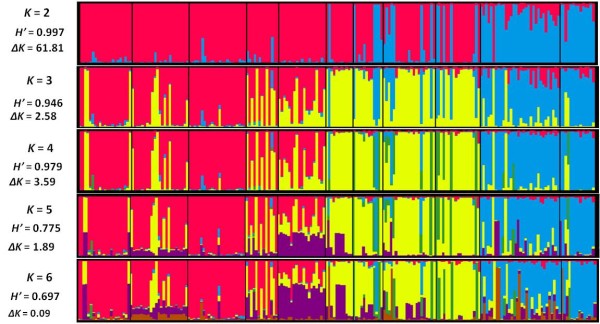
**Genetic structure assessed by STRUCTURE analysis. **Bar plot, generated by DISTRUCT, depicts classifications with the highest probability among assumed clusters in the Mediterranean apricot germplasm. Each individual is represented by a vertical bar, partitioned into colored segments representing the proportion of the individual’s genome in the *K* clusters. Apricot geographic groups were separated by black line.

Using the model-based Bayesian clustering approach implemented in STRUCTURE [45], the genetic structure of Mediterranean apricot was examined according to the model with 2 clusters (*K* = 2) to 6 clusters (*K* = 6). The *ad hoc* quantity based on the second order rate of change of the likelihood function (*ΔK*) [46] revealed a first level of clustering at *K* = 2 for the investigated Mediterranean apricots (*ΔK* = 61.81; Figure 3; see Additional file 4: Figure S1) and a sub-clustering at *K* = 4 (*ΔK* = 3.6). Based on the permuted average Q-matrix generated by CLUMPP for the 10 STRUCTURE runs, the highest similarity coefficient (*H’*) was observed for *K* = 2 (*H’* = 0.997) and *K* = 4 (*H’* = 0.979), indicating the stability of the results for these two models (Figure 3).

At *K* = 2, apricot accessions from Iran and Turkey and a few accessions from Italy and France were separated from the other ones (Figure 3). At *K* = 3, accessions from Continental Europe, South Italy, South France and Murcia were separated from those located in the Maghreb. At *K* = 4, a fourth cluster, including some accessions from Continental Europe, South Italy and South France, was identified as originating from the ‘Adaptive Diversity’ group previously defined by Hagen et al. [35]. At *K* = 5 and *K* = 6, the genetic structure of Mediterranean apricot within three main gene pools was not modified since the accessions of the fifth and sixth clusters were not consistently assigned and hence no distinctive additional cluster was noted (Figure 3).

The *K* = 4 model was chosen to obtain an in-depth overview of apricot genetic structure in the Mediterranean area. Four genetic clusters were thus defined and most of the apricot accessions (167 genotypes among the 207 studied, 80.7%) were assigned to a cluster with a probability superior to 80%: cluster 1 (*blue*) containing 39 accessions originating from the ‘Irano-Caucasian’ area, cluster 2 (*green*) including 7 accessions referred to the ‘Adaptive Diversity’ group [35], cluster 3 (*yellow*) composed of 58 accessions from the ‘North Mediterranean Basin’ area and a few from the South Mediterranean and cluster 4 (*red*) with 63 accessions originating from the ‘South Mediterranean Basin’ region (Figures 1 and 3). The remaining 40 accessions (19.3%) of the sample were assumed to have an admixed ancestry (see Additional file 2: Table S2). The admixture was clearly observed in apricots from the North Tunisia (68.4%) and Moulouya Valley (30.7%) groups (see Additional file 5: Table S4). The four genetic clusters were significantly differentiated, as reflected by the high global F_*ST*_ value (F_*ST*_ = 0.122). The genetic differentiation among the three main clusters 1, 3 and 4 ranged from 0.102 to 0.118, with an average value of 0.109 (see Additional file 6: Table S5).

### A significant reduction of genetic diversity between apricot gene pools

A significant reduction of allelic richness and private allelic richness was observed when regions B and C were compared to region A (Table 4). These observations confirmed the presence of a substantial gradient of decreasing genetic diversity of apricot germplasm from the east (Iran-Caucasian area, region A) to the southwest (Maghreb area, region C), depicting apricot domestication and its diffusion history towards the Mediterranean area [35].

**Table 4:** Relative reduction of diversity among geographic regions and genetic clusters

	***H***_***o***_	***H***_***e***_	***A***_***r***_	***A***_***pr***_
Region A *vs. *B	−0.047	0.034	0.223*	0.573***
Region A *vs. *C	0.182	0.118	0.250**	0.568***
Region B *vs. *C	0.218**	0.087	0.034	−0.012
Cluster 1 *vs. *3	0.060^1^ / 0.051^2^	0.130^1^ / 0.133^2^	**0.303**^1^*** / 0.475^2^***	**0.829**^1^*** / 0.931^2^***
Cluster 1 *vs. *4	**0.238**^1^* / 0.236^2^*	**0.222**^1^* / 0.220^2^*	**0.303**^1^*** / 0.409^2^***	**0.726**^1^*** / 0.832^2^***
Cluster 3 *vs. *4	0.190^1^ / 0.195^2^	0.106^1^ / 0.100^2^	0.000^1^ / -0.125^2^	−0.600^1^ / -1.448^2^

*H*_*o *_observed heterozygosity; *H*_*e *_expected heterozygosity; *A*_*pr *_private allelic richness.

For each estimator, the relative reduction of diversity was determined by calculating 1-(*DIV1*/*DIV2*), where *DIV1* is the estimator of diversity in the supposed original gene pool and *DIV2* is the estimator of diversity in the supposed original gene pool.

Bold values represent significant reduction of diversity at ^*^P < 0.05, ^**^P < 0.01, and ^***^P < 0.001.

^1 ^computed based on all SSR loci used in this study (25 loci); ^2^ computed based only on 24 SSR loci under the assumption of neutrality.

**Figure 4:**
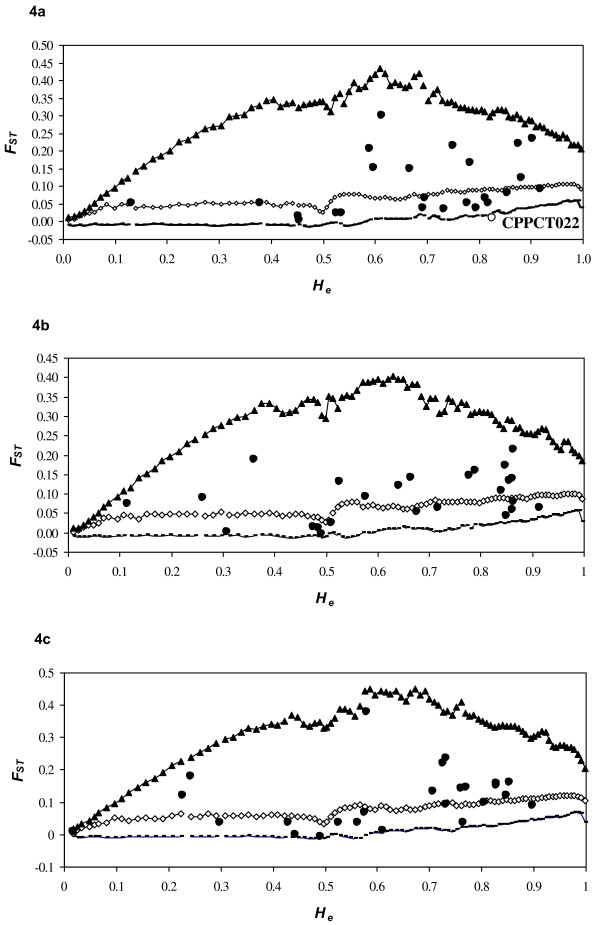
**Simulated F**_**ST **_**values as a function of the expected heterozygosity (*H***_**e**_**) using the F**_**ST **_**between clusters 1 and 3 (*F***_***ST***_ **= 10.5%; a), 1 and 4 (*F***_***ST***_ **= 10%; b), and 3 and 4 (*F***_***ST***_ **= 11.8%; c). **Curves delimiting the neutral expectations with the infinite allele model were computed as described by Beaumont and Nichols [66]. Curves with broken lines, triangles and squares represent the 0.5 (1 – 0.95), 0.5 (1 + 0.95) quantiles and median values, respectively. Black and white circles represent the observations non-significant and significant at 5%, respectively.

To properly assess the reduction of genetic diversity due to the domestication bottleneck, a neutral subsample of loci was determined by removing those presumed to be under selection within populations. Thus, we analysed clusters as defined by a model-based Bayesian clustering since they were significantly differentiated without genetic structure at the intra-cluster level in order to detect true positive outlier loci, as proposed by Excoffier et al. [47]. An analysis using Fdist2 software was conducted on three comparisons of these three main clusters: (*i*) clusters 1 (‘Irano-Caucasian’) and 3 (‘North Mediterranean Basin’), (*ii*) clusters 1 and 4 (‘South Mediterranean Basin’), and (*iii*) clusters 3 and 4 (Figure 4). The F_*ST*_ calculated by Fdist2 between clusters 1 and 3 was 0.105. Based on the first analysis, only one outlier locus was detected at the 95% level: CPPCT022 locus (Figure 4a). This outlier was removed for a second analysis. No outlier was detected and the F_*ST*_ value was 0.110. A similar analysis was carried out on clusters 1 and 4. The F_*ST*_ was 0.100 and no outlier was detected at the 95% level (Figure 4b). Finally, using the same procedure, the F_*ST*_ between clusters 3 and 4 was 0.118. No outlier was detected at the 95% level (Figure 4c).

When looking for the genetic diversity parameters within the three main clusters, similar results were obtained based on both all SSR loci and only on 24 SSR loci following the assumption of neutrality (i.e. after removing the CPPCT022 locus; Table 5). Significant differences were noted for the allelic richness and private allelic richness when comparing clusters 1 and 3 as well as clusters 1 and 4. Further, the observed and expected heterozygosity values were significantly different when comparing clusters 1 and 4 (Table 5). Hence, a significant reduction of allelic richness and private allelic richness was observed using both all SSR loci, and only SSR loci based on the neutrality assumption, when both clusters 3 and 4 were compared to cluster 1 (Table 4). Such a reduction of allelic richness (from 41 to 47%), private allelic richness (from 83 to 93%), and observed and expected heterozygosity (from 22 to 24%) confirmed the decrease in genetic diversity among genetic clusters from the eastern (cluster 1) to the south-western (cluster 4) Mediterranean Basin (Table 4).

**Table 5:** Genetic diversity within the three main clusters identified by the STRUCTURE analysis at *K* = 4^a^

	**Cluster size**	***H***_***o***_^**1**^**/ *H***_***o***_^**2**^	***H***_***e***_^**1**^**/ *H***_***e***_^**2**^	***A***_***r***_^**1,c**^**/ *A***_***r***_^**2,c**^	***A***_***pr***_^**1,c**^**/ *A***_***pr***_^**2,c**^
Cluster 1	39	0.583 / 0.572	0.653 / 0.645	7.747 / 7.625	2.954 / 2.961
Cluster 3	58	0.548 / 0.543	0.568 / 0.559	4.164 / 4.004	0.198 / 0.203
Cluster 4	63	0.444 / 0.437	0.508 / 0.503	4.527 / 4.505	0.504 / 0.497
*P* (Cluster 1 *vs.* 3)		0.621 / 0.757	0.069 / 0.073	10^-6$$$^ / 8.3 10^-7$$$^	4 10^-9$$$^ / 4.7 10^-9$$$^
*P* (Cluster 1 *vs.* 4)		0.037^$^ / 0.048^$^	0.018^$^ / 0.027^$^	7 10^-6$$$^ / 1.6 10^-5$$$^	9 10^-9$$$^ / 9 10^-9$$$^
*P* (Cluster 3 *vs.* 4)		0.055 / 0.059	0.388 / 0.458	0.409 / 0.332	0.008^$$^ / 0.008^$$^

^a ^Cluster 2 was not taken into account in the genetic diversity analysis because of its limited size (seven individuals; Figure 2).

*H*_*o *_observed heterozygosity; *H*_*e *_expected heterozygosity; *A*_*r *_allelic richness; *A*_*pr *_private allelic richness.

^1 ^computed based on all SSR loci used in this study (25 loci); ^2^ computed based only on 24 SSR loci under the assumption of neutrality.

^c ^standardized at maximum value G = 53 individuals per cluster.

Probability of independency between two clusters using the two-tailed Mann-Whitney’s U test. ^$^*P* < 0.05; ^$$^*P* <0.01; ^$$$^*P* < 0.001.

### Specific alleles within pairs of geographic regions and genetic clusters

The number of shared alleles specifically detected between pairs from each geographic region as well as between each cluster pair at all microsatellites was computed. Nineteen alleles observed at 14 SSR loci among the total of 257 alleles detected at the 25 loci (7.4%) and 26 alleles observed at 16 SSR loci (9.7%) were specifically detected within regions A *vs.* B and A *vs.* C, respectively, while only 4 alleles observed at 3 SSR loci were detected within regions B *vs.* C (Table 6). A total of 161 alleles were shared by at least two of the three geographic regions. Among them, 11.8% and 16.1% were specific to regions A *vs.* B and A *vs.* C, respectively, while only 2.4% were specific to regions B *vs.* C (Table 6). The frequency of these alleles ranged from 0.005, corresponding to one allele detected once in each of the two regions, to 0.162, with an average of 0.027 (Table 6). The frequency of these alleles varied according to the locus was higher in region A than B and C when taking all observed shared alleles into account (seeAdditional file 7: Figure S2).

**Table 6:** Specific alleles at each microsatellite locus within geographic region pairs

	**Total alleles**^**1**^	**Shared alleles**^**2**^	**Region A *vs. *B**		**Region A *vs. *C**		**Region B *vs. *C**	
			**Total alleles**^**a**^	**Specific alleles**^**b**^	**Total alleles**^**a**^	**Specific alleles**^**b**^	**Total alleles**^**a**^	**Specific alleles**^**b**^
AMPA109	8	4	7	209 (0.022)^c^	6		7	
CPPCT034	9	7	8		9	193 (0.005)^c^, 207 (0.022)^c^	8	
UDP96-018	6	2	5	263 (0.039)^c^	5		4	
AMPA116	11	7	10	127 (0.162)^c^	11		8	
BPPCT001	5	2	4		5		3	
BPPCT004	12	10	12	176 (0.022)^c^	12	209 (0.022)^c^	10	201 (0.012)^c^
BPPCT030	8	6	7	142 (0.056)^c^	7	150 (0.012)^c^	8	
AMPA101	8	5	7	206 (0.031)^c^	6		7	
AMPA119	8	5	8	114 (0.007)^c^	8	104 (0.014)^c^	5	
BPPCT040	10	4	8	138 (0.019)^c^, 142 (0.014)^c^	9		7	
UDP97-402	10	7	10		8	118 (0.007)^c^, 124 (0.007)^c^, 144 (0.005)^c^	9	
AMPA105	12	6	10	198 (0.072)^c^	10		10	
BPPCT017	10	5	9		9	199 (0.010)^c^, 207 (0.014)^c^	7	
BPPCT038	13	9	11		12		12	135 (0.005)^c^
AMPA100	9	7	9		8	210 (0.029)^c^	8	
BPPCT008	12	9	10	113 (0.014)^c^, 119 (0.012)^c^	11	125 (0.060)^c^, 129 (0.005)^c^	12	
BPPCT025	9	5	8		8		7	
CPPCT030	16	9	12	165 (0.005)^c^	16	169 (0.022)^c^, 173 (0.012)^c^	13	
Ma014a	6	3	5		6	136 (0.007)^c^	4	
Ma040a	11	5	7		10	213 (0.029)^c^	10	
UDP98-412	11	8	10	107 (0.005)^c^, 113 (0.007)^c^	11	93 (0.019)^c^	9	
CPPCT022	15	11	14	234 (0.010)^c^, 246 (0.022)^c^, 262 (0.012)^c^	14	232 (0.007)^c^, 254 (0.022)^c^	13	
CPPCT033	9	5	8	139 (0.056)^c^	8	141 (0.019)^c^, 161 (0.036)^c^	7	
CPPCT006	11	7	10		10	197 (0.155)^c^	9	
UDP98-409	18	13	15		17	122 (0.056)^c^, 130 (0.005)^c^, 150 (0.007)^c^	17	154 (0.089)^c^, 158 (0.005)^c^
Total	257	161	215	19 (0.074)^d^	241	26 (0.097)^d^	196	4 (0.015)^d^

^1 ^total allele observed at each locus.

^2 ^alleles shared at least by two of the three geographic regions.

^a ^alleles observed in each region pairwise (regions A *vs.* B, A *vs.* C and B *vs. *C).

^b ^specific alleles observed in each region pairwise (regions A *vs.* B, A *vs. *C and B *vs. *C).

^c ^frequency of alleles based on the total number of alleles observed at the 25 SSR loci in the 207 apricot accessions studied.

^d ^frequency of specific alleles based on the total number of alleles detected at the 25 loci.

Similar results were obtained when comparing clusters 3 and 4 to cluster 1. Twenty-three alleles observed at 11 SSR loci among the total of 239 alleles detected at the 25 loci (9.6%) and 32 alleles observed at 18 SSR loci (13.3%) were specifically detected within clusters 1 *vs.* 3 and 1 *vs.* 4, respectively; while only one allele observed at locus UDP98-409 was detected within clusters 3 *vs.* 4 (see Additional file 8: Table S6). A total of 141 alleles was shared by at least two of the three genetic clusters. Among them, 16.3% and 22.6% were specific to clusters 1 *vs.* 3 and 1 *vs.* 4, respectively, while only 0.07% were specific to clusters 3 *vs.* 4 (see Additional file 8: Table S6). These results suggest that in the Mediterranean Basin apricot originated from the ‘Irano-Caucasian’ area (region A/cluster 1) and diffused via two different routes, i.e. northern (region B/cluster 3) and southern (region C/cluster 4) routes within the Mediterranean Basin.

## Discussion

### Three main gene pools in the Mediterranean apricot germplasm

Apricot has been mainly cultivated in the Mediterranean area since its earliest introduction [24], while displaying high genetic diversity, as previously reported [27-30]. In this study, analysis of the genetic structure of a large representative sample of apricots located throughout the Mediterranean countries generated new insight into the history of domestication and diffusion of this species within the Mediterranean Basin. Our apricot sample included 207 accessions from Algeria, France, Iran, Italy, Morocco, Spain, Tunisia and Turkey. Apart from apricots from Algeria, Morocco and Tunisia that were sampled *in situ*, all the remaining accessions originated from several *ex situ* collections. Unlike a study on sweet cherry [19] where the authors compared landraces to modern varieties in order to assess the breeding bottleneck, we decided to exclude accessions derived from breeding programs and with unknown passport data in order to obtain clear knowledge about the geographic origin of the studied material and hence to retain only presumed selected local apricots from seed-propagated populations for this study. Despite their ex-situ status, these accessions can be considered as “*in-situ* sampled” since they were originally from specific geographical areas where local apricots have been diversified through selection from seed-propagated populations, as previously mentioned [24]. Using model-based Bayesian clustering without prior information about the geographic origin of the accessions, we obtained a genetic structure pattern similar to those defined with the geographic origin of the accessions. In fact, the distinction of three main genetic clusters [i.e. cluster 1 (‘Irano-Caucasian’ area; in *blue*), cluster 3 (‘North Mediterranean Basin’ area; in *yellow*) and cluster 4 (‘South Mediterranean Basin’ area; in *red*)] by STRUCTURE analysis (Figures 1 and 3) was in concordance with the three regions (A, B and C) defined according to the geographic origin of the accessions, reflecting a long process of apricot domestication and diffusion in the Mediterranean area.

### Loss of genetic diversity supporting the apricot domestication bottleneck

For most crop species, domestication processes cause a loss of genetic diversity due to the bottleneck effect and genetic drift [10-12]. In our study, this loss could be assessed by comparison of levels of diversity between geographic groups or the genetic clusters defined by STRUCTURE analysis (clusters 1 *vs.* 3, clusters 1 *vs.* 4 and clusters 3 *vs.* 4). However, estimation of genetic diversity reduction due to bottleneck domestication could be biased by human selection [9] and the genetic structure within populations [47]. Therefore, we assessed genetic diversity within clusters defined by STRUCTURE using all SSR markers as well as the 24 presumed neutral loci after removing the CPPCT022 outlier locus. This locus was located on linkage group 7 of the *Prunus* genome and not linked to the self-incompatibility locus [48].

The results obtained using the two sets of markers (all SSRs and 24 SSRs under the assumption of neutrality) were congruent for all diversity estimators. A substantial decrease in genetic diversity was observed from the eastern (cluster 1 = ‘Iran-Caucasian’) to the western Mediterranean Basin (cluster 3 = ‘North Mediterranean Basin’ and cluster 4 = ‘South Mediterranean Basin’). Such a loss of genetic diversity was significant when comparing clusters 1 (‘Iran-Caucasian’) and 3 (‘North Mediterranean Basin’) and clusters 1 and 4 (‘South Mediterranean Basin’). Nevertheless, it was not significant when cluster 3 was compared to cluster 4, despite the different modes of apricot propagation: vegetative in North Mediterranean *vs.* sexual reproduction in South Mediterranean, especially in the oasis agroecosystems in the Maghreb area [33,34].

We thus noted a substantial loss of genetic diversity that was independent of the selection impact due to the domestication bottleneck. Such a loss of genetic diversity is closely in line with the apricot diffusion routes, and contrasting with patterns in other native Mediterranean fruit species such as olive and grape for which a weak loss of genetic diversity between varieties and wild relatives has been observed [17,18]. The magnitude of the bottleneck depends on the number of individuals involved and the duration of these pressures [13]. In sweet cherry, several successive domestication events have probably occurred and a significant bottleneck associated with modern breeding was revealed, while no reduction in diversity has been shown between landraces and wild relatives [19].

### Two routes of apricot diffusion into the Mediterranean Basin

The Irano-Caucasian area is considered as a secondary diversification zone [24] for at least two reasons. First, based on ethno-botanical data, northern Iran was identified as an evolutionary centre for a large number of fruit trees, including *Prunus* species [49]. Second, Irano-Caucasian apricots occupy an intermediate position between domesticated varieties and wild species, as previously described [24]. Similar findings were also obtained for Iranian apples [50]. Although we lacked genetic data from the presumed primary gene pool of the centre of apricot origin in China [22,23], our findings suggest that Mediterranean apricots have been selected from the ‘Irano-Caucasian’ gene pool. Indeed, 62% of alleles common to the three regions A, B and C and revealed in the northern and southern Mediterranean Basin were found to be shared with the ‘Irano-Caucasian’ gene pool. This leads to the following question: did these two gene pools diffuse into the Mediterranean Basin through only one route, as proposed by Kostina [25], or two main routes, i.e. via the northern and southern Mediterranean, as hypothesised by Faust et al. [24] and Mehlenbacher et al. [26]?

The distinction of ‘South-Mediterranean’ apricots from the ‘Irano-Caucasian’ cluster confirmed the findings of a previous study [22] based on morphological characters. Moreover, according to our analyses, most accessions from the Murcia group (Spain) were assigned to region C, but it seems that this group is genetically intermediate between regions B (Continental Europe, South France and South Italy groups) and C (South Mediterranean). Spanish and North African accessions were also pooled in another AFLP-based study [35]. In fact, the introduction of apricots into North Africa and Spain was attributed to the Arabs during the regime of Umayyad, who conquered Spain between 711 and 719 [24]. Furthermore, region C pooled apricots propagated by grafting (from Murcia and North Tunisia) and by seeds (from the oasis agroecosystems: Moulouya Valley, Messaad, Oases of Tunisia and Draa Valley). These two apricot groups, also distinct according to their mode of propagation, were recently proved to share a common genetic basis in Tunisia [34]. In addition, some accessions encountered in North Tunisia, Messaad and Moulouya Valley represented a clear admixture between clusters 3 and 4, indicating that gene exchanges have occurred between northern and southern Mediterranean countries. These events could be related to both ancient human movements (e.g. Romans, Arabs, Andalusians) and/or to recent material transfers associated with the French colonisation period in the early 20^th^ century [24,26].

By comparing cluster pairs 1 *vs.* 3 and 1 *vs.* 4, we observed a significant loss of genetic diversity. Conversely, we noted no significant loss of genetic diversity between clusters 3 *vs.* 4, while they were more differentiated than cluster pairs 1 *vs.* 3 and 1 *vs.* 4 (see Additional file 6: Table S5). Otherwise, a substantial proportion of specific alleles was observed along the northern Mediterranean apricot diffusion route (clusters 1 *vs.* 3 and geographic group A *vs.* B) and the southern route (clusters 1 *vs.* 4 geographic group A *vs.* C). These results strongly indicate that apricot was diffused through two main routes: the first one through countries north of the Mediterranean Sea (cluster 3/region B) and the second one probably brought by the Arabs through North African countries (cluster 4/region C), as previously proposed [51,52]. Our findings are in agreement with earlier studies [24,26], but not with the hypothesis proposed by Kostina [25], who reported only one major route from the Irano-Caucasian area to the Mediterranean Basin.

## Conclusions

Based on the three main identified gene pools, we observed a significant and substantial loss of apricot genetic diversity, ranging from about 37 to 49% from the secondary apricot diversification zone (‘Irano-Caucasian’) to the southwestern Mediterranean Basin, depicting a genetic signature of apricot domestication and diffusion into the Mediterranean Basin. Unlike Kostina’s assumptions [25], we propose an evolutionary scenario in favour of two diffusion routes in southern Europe and North Africa as revealed by a substantial proportion of shared alleles that were specifically detected along each of the two diffusion routes. Our study generated genetic insight to: (*i*) improve management and conservation strategies for Mediterranean apricot germplasm, and (*ii*) propose a genetic basis for apricot breeding programs.

## Methods

### Plant material

A total of 207 apricot accessions were sampled throughout different countries from the eastern to the south-western Mediterranean Basin: Algeria (23), France (49), Italy (19), Morocco (34), Spain (8), Tunisia (42) and Turkey (32; Figure 1; see Additional file 2: Table S2). The strategy was to select accessions reflecting the local variability in each country, excluding accessions derived from breeding programs. According to the surveyed country, apricot material was collected either from germplasm collections, including the French collection maintained at the *Institut National de la Recherche Agronomique* (INRA Avignon, France), the Italian collection of the Department of Fruit Science and Plant Protection of Woody Species (University of Pisa, Italy), the Spanish collection of CEBAS-CSIC (Murcia, Spain), and the Turkish collection of the Inonu University (Malatya, Turkey), or from different collection surveys as was the case for the Algerian, Moroccan and Tunisian accessions. The French material studied contained native cultivars as well as a few introduced accessions initially acquired from other collections around the world (Italy, Iran, Spain), which were also considered in the sample set in order to span a broad eco-geographical apricot distribution range (Figure 1; see Additional file 2: Table S2). Apricots are vegetatively propagated, however traditional local cultivars propagated by grafting and accessions propagated by seeds grown in oasis agroecosystems were both present in North African countries.

The studied accessions were subdivided into eleven groups based on their geographic origin (Figure 1; see Additional file 2: Table S2). Group 1 (Iran) consisted of 14 Iranian varieties present in the French collection, group 2 (Turkey) was composed of 32 accessions from Turkey, group 3 (Continental Europe) was composed of 21 accessions from both northern Italy and northern France, since these two populations are known to be related to a Central Europe gene pool, which is less adapted to the Mediterranean region [24], group 4 (South France) comprised 12 accessions originating from the southern regions of France, group 5 (South Italy) included 18 accessions from the southern Italy and specifically the Napoli area, group 6 (Murcia) consisted of 11 accessions from Murcia in Spain, group 7 (North Tunisia) consisted of 19 grafted propagated cultivars collected from Testour and Ras Jbel regions in northern Tunisia, group 8 (Moulouya Valley) encompassed 13 accessions from the Moulouya Valley in central eastern Morocco, group 9 (Messaad) was composed of 23 accessions from Messaad region in Algeria, group 10 (Oases of Tunisia) consisted of 23 seed-propagated apricot accessions collected from five different oases in Tunisia (i.e. Tameghza, Nefta, Tozeur, Midess and Degache), and finally group 11 (Draa Valley) included 21 accessions from the Draa Valley in south-eastern Morocco.

### DNA extraction and microsatellite analysis

Except for the Turkish accessions, all the remaining material shipped to the INRA Montpellier laboratory for DNA extraction were in the form of fresh young leaves collected in each country of origin during the apricot flowering period (between March and May 2008). For these latter, total genomic DNA extraction was conducted from 150 mg of fresh young leaves, using the DNeasy Plant Mini Kit (QIAGEN, Germany) according to the manufacturer's instructions, with minor modifications: addition of 1% w/v of PVP-40 to the AP1 buffer solution. For the Turkish accessions, total genomic DNA was extracted at the Apricot Research Center of the Inonu University of Malatya according to the same protocol. Then DNA aliquots were purified in the INRA Montpellier laboratory according to the protocol described above.

Twenty-five microsatellite markers were selected on the basis of the ease of amplification in apricot and their location on the *Prunus* reference genetic map: ‘Texas’ x ‘Earlygold’ [53,54] as they are evenly distributed throughout the eight linkage groups of the *Prunus* genome (Table 1). These SSR loci were successfully used in the study on Tunisian apricot [34] and Mnejja et al. [36] have demonstrated the successful transferability of most of them among *Prunus* species. PCR was carried out in a 20 μl reaction mixture, containing 20 ng of template DNA, 2 mM MgCl2, 4 pmol of reverse primer and 1 pmol of forward primer, 0.2 mM of each dNTP, and 1 U of *Taq* polymerase (Sigma, USA). The forward primer was 5’-labeled with one of the three fluorochromes (6FAM, NED or HEX). The PCR conditions were as follows: 35 cycles at 94°C for 30 s, T° annealing (depending on the locus) for 60 s, and 72°C for 60 s, with a final extension step at 72°C for 10 min. Amplified products were resolved using an ABI prism 3130 XL automatic DNA sequencer (Applied Biosystems, USA). Allele sizes were determined with GeneMapper 3.7 software (Applied Biosystems, USA).

### Genetic structure analysis

The model-based clustering approach, as implemented in the STRUCTURE 2.2 software program [45], was used to infer the population structure of Mediterranean apricot accessions. This program assumes Hardy–Weinberg equilibrium and linkage equilibrium within clusters. No prior information about the geographic origin of the accessions was considered in the analysis. The STRUCTURE algorithm was run using a model with admixture and correlated allele frequencies, with 10 independent replicate runs for each *K* value (number of genetic clusters), for *K* ranging from 1 to 6. Each run involved a burning period of 100000 iterations, and a post-burning simulation length of 1000000. The run with the maximum likelihood was used to assign the most probable number of clusters, which was validated with an ad hoc statistic based on the second order rate of change in the log probability of data between successive *K* values [46]. To find optimal alignments of independent runs, the CLUMPP version 1.1 software program [55] was used with greedy algorithms, 10,000 random input orders and 10,000 repeats, to calculate the average pairwise similarity (*H’*) of runs. The output obtained was used directly as input by the cluster DISTRUCT version 1.1 visualization program [56].

### Genetic diversity and differentiation assessment

GENETIX 4.05 [57] was used to calculate the following parameters: total number of alleles (*N*_*A*_), number of alleles with frequency higher than 5% (*N*_*A,P*_), and observed (*H*_*o*_) and expected (*H*_*e*_) heterozygosities. PowerMarker 3.25 [58] was used to estimate the polymorphic information content (PIC) at each locus, originally defined as the probability of a given marker being informative in a random mating [59]. The inbreeding coefficient (*F*_*IS*_) and the genetic differentiation (*F*_*ST*_) were computed according to the formula of Weir and Cockerham [60] using GENEPOP 4.0 [61] and Fisher’s method [62] was applied to test the significance of pairwise *F*_*ST*_ values. The generalized rarefaction approach ADZE [63] with standardized values was used to estimate the allelic richness (*A*_*r*_) and the private allelic richness (*A*_*pr*_).

Pairwise standard genetic distances of Nei [44] were calculated and used to conduct cluster analysis with the Neighbor-joining algorithm and to construct an unrooted tree with 10,000 bootstraps over microsatellite loci, as implemented in PHYLIP 3.69 [64]. The analysis of molecular variance (AMOVA) implemented in GENALEX [65] was conducted to estimate the hierarchical differentiation at two levels: (*i*) a group level identifying the eleven apricot geographic groups; and (*ii*) a region level distinguishing the three geographic regions A (Iran and Turkey), B (Continental Europe, South France and South Italy), and C (Murcia, North Tunisia, Moulouya Valley, Messaad, Oases of Tunisia and Draa Valley).

### Sub-samples of neutral markers definition

The observed variation of genetic diversity among clusters might be due to two evolutionary factors: selection and bottleneck effects. Selection force could affect the estimation of bottleneck impact on the apricot diffusion history. A sub-sample of neutral markers was thus defined and used for the genetic diversity analysis. For this purpose, the method developed by Beaumont and Nichols [66] was used, involving the detection of unusually high or low *F*_*ST*_ levels by plotting *F*_*ST*_ against heterozygosity on the set of markers. The Fdist2 analysis was conducted on three comparisons of the three main genetic clusters. We compared (*i*) clusters 1 (‘Irano-Caucasian’) and 3 (‘North Mediterranean Basin’), (*ii*) clusters 1 and 4 (‘South Mediterranean Basin’), and (*iii*) clusters 3 and 4. For each comparison, 50000 simulations were run using the infinite allele model for markers. A first analysis revealed a first set of outliers. Then they were removed and a new *F*_*ST*_ was calculated, which was used to make a new analysis and reveal a new set of outliers. Analyses were iterated until no further locus fell outside of the expected distribution. The last *F*_*ST*_ value was used as the neutral value to detect outliers on the whole set of data.

### Determination of the relative reduction of diversity

The relative reduction of diversity was estimated with five diversity estimators: the total number of alleles, allelic richness, private allelic richness, observed heterozygosity and the expected heterozygosity, as described by Vigouroux et al. [67]. The probability of independency between regions/clusters was determined for each of these diversity estimators by the two-tailed Mann-Whitney’s U test using the R version R 2.11.0 software package [68]. For each estimator, the relative reduction of diversity was determined by calculating 1-(*DIV1*/*DIV2*), where *DIV1* is the estimator of diversity in the supposed derivating genetic pool and *DIV2* is the estimator of diversity in the supposed originating genetic pool. The relative reduction of diversity was estimated using all SSR markers for the comparison among regional groups, and based on all 25 SSR markers and also only on neutral SSR markers when comparing the three main genetic clusters.

## Authors’ contributions

BK designed and coordinated the study, performed the statistical analyses and drafted the manuscript. HB participated in the design of the study, conducted the molecular analysis, participated in the statistical analyses, and drafted the manuscript. JMA supervised the research project, prepared the plant sampling, helped in designing the study and drafting the manuscript. LK, AM, ST, CD and BMA contributed to plant sampling and descriptions of accessions. NTF helped in designing the study and drafting the manuscript. SS supervised the molecular analysis. All authors participated in the finalising the text and approved the final manuscript.

## Authors’ information

HB is a PhD student who will defend her thesis entitled “Apricot genetic structure in the Mediterranean Basin: domestication and diffusion process” in 2012. JMA is a scientist at INRA, the French national institute for agricultural research, and is in charge of INRA’s fruit research group and in particular apricot research program including genetic resource management and breeding programs. LK is an Assistant Professor at the University of Sciences of Gafsa and a scientist in the Laboratory of Molecular Genetics, Immunology and Biotechnology at the University of Sciences of Tunis and is in charge of the apricot genetic resource management. NTF is a Professor at the University of Sciences of Tunis and Director of the Laboratory of Molecular Genetics, Immunology and Biotechnology at the University of Sciences of Tunis and is in charge of fruit genetic resource management. AM is a scientist at the Moroccan national institute for agricultural research and is in charge of apricot genetic resource management and breeding. ST is a scientist at the *Laboratoire d’Arboriculture* at the University of Blida. CD is an Assistant Professor at the University of Pisa, Head of the Laboratory of Molecular Biology of Fruit Science and in charge of local fruit resource genotyping. BMA is a scientist in the Department of Biology at Inonu University. SS is an engineer, molecular biologist, and is in charge of the development and use of molecular markers. BK is a geneticist in the conservation biology field, focusing on diversification of Mediterranean fruit species and on the management of agrobiodiversity and agroecosystems.

## Supplementary Material

Additional file 1 **Table S1. **Genetic diversity at each of the 25 SSR loci used in the three geographic regions. *F*_*IS *_fixation index values; bold values represented exact test significant at *P* < 0.01. Click here for file

Additional file 2 **Table S2. **Apricot germplasm accessions, code, origin as well as geographic group and genetic cluster to which each accession was assigned.^*^in situ sampling. ^a ^The clusters were assigned based on STRUCTURE analysis with cluster 1 = ‘Irano-Caucasian’ (*blue*), cluster 2 = ‘Adaptive Diversity’ (*green*), cluster 3 = ‘North Mediterranean Basin’ (*yellow*) and cluster 4 = ‘South Mediterranean Basin’ (*red*). Some accessions were assigned as mixed clusters because they were assumed to have admixed ancestry (Figure 2). Click here for file

Additional file 3 **Table S3. **Geographic group pairwise comparisons. Nei’s [44] genetic distances (above diagonal) and *F*_*ST *_(below diagonal); Global *F*_*ST*_ = 0.111 ^*^P < 10^-4^; ^**^P < 10^-6^. Click here for file

Additional file 4 **Figure S1. **Description of the four steps for the graphical method allowing determination of optimal *K *according to Evanno’s parameters. Click here for file

Additional file 5 **Table S4. **Information on the number of assigned accessions per geographic group and region. Click here for file

Additional file 6 **Table S5.** Pairwise *F*_*ST *_among the four genetic clusters defined by the STRUCTURE analysis. All *F*_*ST *_values were significant at *P* < 10^-6^; Global *F*_*ST*_ = 0.122; Global *F*_*ST *_among the 3 main clusters = 0.109 significant at *P* < 10^-4^. Click here for file

Additional file 7 **Figure S2. **Histogram illustrating the frequency distributions of alleles for each microsatellite among geographic regions. Arrows indicate specific alleles detected in each geographic region pairwise with A vs. B, A vs. C and B vs. C. Click here for file

Additional file 8 **Table S6. **Specific alleles at each microsatellite locus within genetic cluster pairs.^1^ total allele observed at each locus. ^2 ^alleles shared by at least two of the three clusters. ^a ^alleles observed in each cluster pairwise (clusters 1 *vs. *3, 1 *vs. *4 and 3 *vs*. 4). ^b ^specific alleles observed in each cluster pairwise (clusters 1 *vs. *3, 1 *vs. *4 and 3 *vs. *4). ^c^ frequency of alleles based on the total number of alleles observed at the 25 SSR loci in the 207 apricot accessions studied. ^d ^frequency of specific alleles based on the total number of alleles detected at the 25 loci. Click here for file
