# Supplementary material for: Loss of genetic diversity as a signature of apricot domestication and diffusion into the Mediterranean Basin
Source: BMC Plant Biol. 2012 Apr 17;12:49. doi: 10.1186/1471-2229-12-49 (PMC3511222; doi:10.1186/1471-2229-12-49)
Supplement: Additional file 1 — Table S1. Genetic diversity at each of the 25 SSR loci used in the three geographic regions. FIS fixation index values; bold values represented exact test significant at P < 0.01. [file 1471-2229-12-49-S1.doc]

**Additional file 1. Table S1 – Genetic diversity at each of the 25 SSR loci used in the three geographic regions**

| Locus | Region A : ‘Irano-Caucasian’ | | | |  | Region B : ‘North Mediterranean Basin’ | | | |  | Region C : ‘South Mediterranean Basin’ | | | |
| --- | --- | --- | --- | --- | --- | --- | --- | --- | --- | --- | --- | --- | --- | --- |
|  | *H_e_* | *H_o_* | *F_IS_* | *P*-value |  | *H_e_* | *H_o_* | *F_IS_* | *P*-value |  | *H_e_* | *H_o_* | *F_IS_* | *P*-value |
| CPPCT006 | 0.8266 | 0.8261 | 0.0006 | 0.1915 |  | 0.6956 | 0.6471 | 0.0704 | 0.4061 |  | 0.8072 | 0.6727 | **0.1672** | **0** |
| CPPCT033 | 0.6672 | 0.6522 | 0.0228 | 0.2286 |  | 0.4056 | 0.3922 | 0.0333 | 0.2787 |  | 0.3687 | 0.2909 | **0.2117** | **0.0025** |
| CPPCT034 | 0.4486 | 0.4348 | 0.0312 | 0.0329 |  | 0.3403 | 0.3922 | -0.1541 | 0.8919 |  | 0.5287 | 0.4909 | 0.0718 | 0.5286 |
| AMPA100 | 0.8184 | 0.8261 | -0.0094 | 0.4808 |  | 0.7061 | 0.7647 | -0.0839 | 0.1507 |  | 0.668 | 0.5909 | 0.1158 | 0.0603 |
| AMPA116 | 0.7508 | 0.8261 | -0.1014 | 0.0973 |  | 0.8059 | 0.7647 | 0.0516 | 0.3353 |  | 0.7439 | 0.5909 | 0.2064 | 0.0183 |
| BPPCT004 | 0.8282 | 0.6957 | **0.1616** | **0.0066** |  | 0.7692 | 0.6863 | 0.1087 | 0.257 |  | 0.7579 | 0.6182 | **0.185** | **0** |
| AMPA101 | 0.7179 | 0.6957 | 0.0313 | 0.6316 |  | 0.5999 | 0.6471 | -0.0795 | 0.2915 |  | 0.6333 | 0.5091 | **0.1969** | **0.0068** |
| BPPCT040 | 0.4345 | 0.4348 | -0.0006 | 0.1231 |  | 0.5622 | 0.5686 | -0.0115 | 0.8825 |  | 0.3757 | 0.3364 | 0.1052 | 0.0168 |
| UDP96-018 | 0.2 | 0.2174 | -0.0883 | 1 |  | 0.1817 | 0.1569 | 0.1379 | 0.0175 |  | 0.0181 | 0 | **1** | **0.0046** |
| AMPA105 | 0.7984 | 0.7174 | 0.1024 | 0.3725 |  | 0.794 | 0.7059 | **0.112** | **0.0044** |  | 0.6703 | 0.6727 | -0.004 | 0.6136 |
| BPPCT001 | 0.5268 | 0.4783 | 0.093 | 0.4946 |  | 0.477 | 0.5686 | -0.1944 | 0.238 |  | 0.4033 | 0.4182 | **-0.037** | **0.0037** |
| UDP98-409 | 0.8641 | 0.8261 | 0.0444 | 0.1366 |  | 0.8035 | 0.7843 | 0.0242 | 0.3392 |  | 0.8664 | 0.6818 | **0.2138** | **0** |
| CPPCT022 | 0.8662 | 0.8261 | 0.0468 | 0.288 |  | 0.7917 | 0.7843 | 0.0094 | 0.5133 |  | 0.7325 | 0.6364 | **0.1317** | **0.0037** |
| Ma040a | 0.5067 | 0.3261 | **0.359** | **0.0009** |  | 0.6449 | 0.6078 | **0.058** | **0.0038** |  | 0.6376 | 0.5 | 0.2165 | 0.0116 |
| AMPA109 | 0.2926 | 0.2391 | 0.1845 | 0.1715 |  | 0.5304 | 0.4314 | **0.1882** | **0.0007** |  | 0.1212 | 0.1091 | 0.1001 | 0.3565 |
| CPPCT030 | 0.8258 | 0.8043 | 0.0263 | 0.8319 |  | 0.589 | 0.5882 | 0.0013 | 0.0781 |  | 0.7443 | 0.7273 | 0.0229 | 0.0187 |
| BPPCT008 | 0.7396 | 0.6957 | 0.0601 | 0.63 |  | 0.7391 | 0.7647 | -0.035 | 0.4068 |  | 0.799 | 0.6818 | **0.1472** | **0.0048** |
| BPPCT017 | 0.4252 | 0.3913 | 0.0806 | 0.4114 |  | 0.6137 | 0.7451 | -0.2168 | 0.1573 |  | 0.588 | 0.5455 | **0.0726** | **0.0016** |
| BPPCT025 | 0.7191 | 0.6304 | 0.1245 | 0.7329 |  | 0.5341 | 0.541 | 0.1569 | 0.0811 |  | 0.6012 | 0.5636 | 0.0628 | 0.0238 |
| BPPCT030 | 0.7463 | 0.6957 | 0.0686 | 0.0289 |  | 0.7674 | 0.8235 | -0.0739 | 0.7779 |  | 0.7152 | 0.6545 | 0.0852 | 0.0881 |
| BPPCT038 | 0.7303 | 0.7609 | -0.0424 | 0.7464 |  | 0.7661 | 0.7843 | -0.0241 | 0.2583 |  | 0.7159 | 0.5545 | **0.2262** | **0** |
| UDP98-412 | 0.6417 | 0.5217 | 0.1886 | 0.0798 |  | 0.7655 | 0.8235 | **-0.0766** | **0.0062** |  | 0.6192 | 0.4455 | **0.2815** | **0.0004** |
| AMPA119 | 0.5241 | 0.4348 | 0.172 | 0.2973 |  | 0.5244 | 0.5882 | -0.1232 | 0.8575 |  | 0.1221 | 0.0727 | **0.4054** | **0.0001** |
| Ma014a | 0.6777 | 0.5652 | 0.1676 | 0.3107 |  | 0.505 | 0.4902 | 0.0295 | 1 |  | 0.4719 | 0.4364 | **0.0757** | **0.0048** |
| UDP97-402 | 0.5447 | 0.3913 | **0.2838** | **0.0068** |  | 0.6562 | 0.6471 | 0.014 | 0.0648 |  | 0.509 | 0.4 | 0.2149 | 0.0239 |

*F_IS_* fixation index values; bold values represented exact test significant at *P* < 0.01
