# Supplementary material for: Loss of genetic diversity as a signature of apricot domestication and diffusion into the Mediterranean Basin
Source: BMC Plant Biol. 2012 Apr 17;12:49. doi: 10.1186/1471-2229-12-49 (PMC3511222; doi:10.1186/1471-2229-12-49)
Supplement: Additional file 3 — Table S3. Geographic group pairwise comparisons. Nei’s [44] genetic distances (above diagonal) and FST (below diagonal); Global FST = 0.111 *P < 10-4; **P < 10-6. [file 1471-2229-12-49-S3.doc]

**Additional file 3. Table S3 -** Geographic group pairwise comparisons

|  | Iran | Turkey | Continental Europe | South France | South Italy | Murcia | North Tunisia | Moulouya Valley | Messaad | Oases of Tunisia | Draa Valley |
| --- | --- | --- | --- | --- | --- | --- | --- | --- | --- | --- | --- |
| Iran |  | 0.092 | 0.214 | 0.242 | 0.314 | 0.343 | 0.264 | 0.237 | 0.260 | 0.219 | 0.214 |
| Turkey | 0.024^**^ |  | 0.159 | 0.182 | 0.234 | 0.282 | 0.258 | 0.211 | 0.195 | 0.181 | 0.219 |
| Continental Europe | 0.079^**^ | 0.066^**^ |  | 0.113 | 0.095 | 0.158 | 0.258 | 0.179 | 0.215 | 0.232 | 0.179 |
| South France | 0.086^**^ | 0.071^**^ | 0.037^**^ |  | 0.187 | 0.264 | 0.273 | 0.225 | 0.284 | 0.303 | 0.265 |
| South Italy | 0.129^**^ | 0.106^**^ | 0.037^**^ | 0.084^**^ |  | 0.245 | 0.276 | 0.252 | 0.244 | 0.240 | 0.219 |
| Murcia | 0.132^**^ | 0.120^**^ | 0.065^**^ | 0.112^**^ | 0.118^*^ |  | 0.186 | 0.234 | 0.243 | 0.340 | 0.292 |
| North Tunisia | 0.119^**^ | 0.123^**^ | 0.089^**^ | 0.131^**^ | 0.144^**^ | 0.094^**^ |  | 0.160 | 0.188 | 0.211 | 0.172 |
| Moulouya Valley | 0.102^**^ | 0.100^**^ | 0.085^**^ | 0.105^**^ | 0.131^**^ | 0.114^**^ | 0.086^**^ |  | 0.221 | 0.181 | 0.095 |
| Messaad | 0.139^**^ | 0.111^**^ | 0.124^**^ | 0.158^**^ | 0.147^**^ | 0.145^**^ | 0.122^**^ | 0.140^**^ |  | 0.137 | 0.148 |
| Oases of Tunisia | 0.123^**^ | 0.107^**^ | 0.136^**^ | 0.171^**^ | 0.148^**^ | 0.195^**^ | 0.138^**^ | 0.118^**^ | 0.103^**^ |  | 0.109 |
| Draa Valley | 0.121^**^ | 0.127^**^ | 0.109^**^ | 0.153^**^ | 0.138^**^ | 0.173^**^ | 0.115^**^ | 0.053^**^ | 0.111^**^ | 0.082^**^ |  |

Nei’s [44] genetic distances (above diagonal) and *F_ST_* (below diagonal); Global *F_ST_* = 0.111

^*^ *P* < 10^-4^; ^**^ *P* < 10^-6^
