# Supplementary material for: Loss of genetic diversity as a signature of apricot domestication and diffusion into the Mediterranean Basin
Source: BMC Plant Biol. 2012 Apr 17;12:49. doi: 10.1186/1471-2229-12-49 (PMC3511222; doi:10.1186/1471-2229-12-49)
Supplement: Additional file 4 — Figure S1. Description of the four steps for the graphical method allowing determination of optimal K according to Evanno’s parameters. [file 1471-2229-12-49-S4.doc]

Mean L(K)

800

-14500

-13500

-13000

-12500

-12000

Mean L’’(K)

0

20

40

60

1

2

3

4

5

6

0

50

100

150

200

250

1

2

3

4

5

6

0

200

400

600

1

2

3

4

5

6

-14000

1

2

3

4

5

6

K

K

Mean DeltaK

K

K

Mean L’(K)

**Additional file 4. Figure S1 - Description of the four steps for the graphical method allowing determination of optimal *K* according to Evanno’s parameters**
