# Supplementary material for: Loss of genetic diversity as a signature of apricot domestication and diffusion into the Mediterranean Basin
Source: BMC Plant Biol. 2012 Apr 17;12:49. doi: 10.1186/1471-2229-12-49 (PMC3511222; doi:10.1186/1471-2229-12-49)
Supplement: Additional file 5 — Table S4. Information on the number of assigned accessions per geographic group and region. [file 1471-2229-12-49-S5.doc]

**Additional file 5. Table S4 - Information on the number of assigned accessions per geographic group and** region

| Groups | Total number of accessions | Number of assigned accessions | Number of admixed accessions |
| --- | --- | --- | --- |
| Iran | 14 | 12 | 2 |
| Turkey | 32 | 26 | 6 |
| **Region A** | **46** | **38** | **8** |
| Continental Europe | 21 | 17 | 4 |
| South France | 12 | 12 | 0 |
| South Italy | 18 | 15 | 3 |
| **Region B** | **51** | **44** | **7** |
| Murcia | 11 | 11 | 0 |
| North Tunisia | 19 | 6 | 13 |
| Moulouya Valley | 13 | 9 | 4 |
| Messaad | 23 | 18 | 5 |
| Oases of Tunisia | 23 | 21 | 2 |
| Draa Valley | 21 | 20 | 1 |
| **Region C** | **110** | **85** | **25** |
