# Supplementary material for: Loss of genetic diversity as a signature of apricot domestication and diffusion into the Mediterranean Basin
Source: BMC Plant Biol. 2012 Apr 17;12:49. doi: 10.1186/1471-2229-12-49 (PMC3511222; doi:10.1186/1471-2229-12-49)
Supplement: Additional file 6 — Table S5. Pairwise FST among the four genetic clusters defined by the STRUCTURE analysis. All FST values were significant at P < 10-6; Global FST = 0.122; Global FST among the 3 main clusters = 0.109 significant at P < 10-4. [file 1471-2229-12-49-S6.doc]

**Additional file 6. Table S5 - Pairwise *F_ST_* among the four genetic clusters defined by the STRUCTURE** analysis

|  | Cluster 1 (N = 39) | Cluster 2 (N = 7) | Cluster 3 (N = 58) |
| --- | --- | --- | --- |
| Cluster 2 (N = 7) | 0.176 |  |  |
| Cluster 3 (N = 58) | 0.106 | 0.205 |  |
| Cluster 4 (N = 63) | 0.102 | 0.244 | 0.118 |

All *F_ST_* values were significant at *P* < 10^-6^; Global *Fst* = 0.122; Global *Fst* among the 3 main clusters = 0.109 significant at *P* < 10^-4^
