# Supplementary material for: Loss of genetic diversity as a signature of apricot domestication and diffusion into the Mediterranean Basin
Source: BMC Plant Biol. 2012 Apr 17;12:49. doi: 10.1186/1471-2229-12-49 (PMC3511222; doi:10.1186/1471-2229-12-49)
Supplement: Additional file 7 — Figure S2. Histogram illustrating the frequency distributions of alleles for each microsatellite among geographic regions. Arrows indicate specific alleles detected in each geographic region pairwise with A vs. B, A vs. C and B vs. C. [file 1471-2229-12-49-S7.doc]

**Additional file 7. Figure S2 – Histogram illustrating the frequency distributions of alleles for each microsatellite among geographic regions. Arrows indicate specific alleles detected in each geographic region pairwise ( A *vs.* B, A *vs.* C and B *vs.* C)**
