# Supplementary material for: Loss of genetic diversity as a signature of apricot domestication and diffusion into the Mediterranean Basin
Source: BMC Plant Biol. 2012 Apr 17;12:49. doi: 10.1186/1471-2229-12-49 (PMC3511222; doi:10.1186/1471-2229-12-49)
Supplement: Additional file 8 — Table S6. Specific alleles at each microsatellite locus within genetic cluster pairs.1 total allele observed at each locus. 2 alleles shared by at least two of the three clusters. a alleles observed in each cluster pairwise (clusters 1 vs. 3, 1 vs. 4 and 3 vs. 4). b specific alleles observed in each cluster pairwise (clusters 1 vs. 3, 1 vs. 4 and 3 vs. 4). c frequency of alleles based on the total number of alleles observed at the 25 SSR loci in the 207 apricot accessions studied. d frequency of specific alleles based on the total number of alleles detected at the 25 loci. [file 1471-2229-12-49-S8.doc]

**Additional file 8. Table S6** – Shared alleles for all the 25 microsatellite loci among clusters

| Locus | Total alleles^1^ | Shared alleles^2^ | Cluster 1 *vs.* Cluster 3 | | Cluster 1 *vs.* Cluster 4 | | Cluster 3 *vs.* Cluster 4 | |
| --- | --- | --- | --- | --- | --- | --- | --- | --- |
|  |  |  | Total alleles^a^ | Specific alleles^b^ | Total alleles^a^ | Specific alleles^b^ | Total alleles^a^ | Specific alleles^b^ |
| AMPA109 | 7 | 3 | 6 |  | 7 | 199 (0.015)^c^ | 4 |  |
| CPPCT034 | 8 | 5 | 7 |  | 8 | 189 (0.009)^c^, 199 (0.015)^c^, 207 (0.021)^c^ | 6 |  |
| UDP96-018 | 5 | 1 | 5 |  | 4 |  | 2 |  |
| AMPA116 | 10 | 7 | 10 |  | 10 | 115 (0.056)^c^, 137 (0.009)^c^ | 7 |  |
| BPPCT001 | 5 | 2 | 5 |  | 5 |  | 2 |  |
| BPPCT004 | 12 | 10 | 12 | 176 (0.026)^c^, 178 (0.018)^c^ | 12 | 201 (0.012)^c^, 203 (0.065)^c^, 209 (0.021)^c^ | 10 |  |
| BPPCT030 | 8 | 6 | 8 | 142 (0.046)^c^ | 8 | 150 (0.012)^c^ | 6 |  |
| AMPA101 | 7 | 4 | 6 | 194 (0.059)^c^ | 7 |  | 5 |  |
| AMPA119 | 8 | 5 | 7 | 98 (0.125)^c^ | 8 | 104 (0.018)^c^ | 4 |  |
| BPPCT040 | 9 | 2 | 8 |  | 9 |  | 3 |  |
| UDP97-402 | 9 | 4 | 9 |  | 9 | 124 (0.009)^c^ | 4 |  |
| AMPA105 | 11 | 5 | 9 |  | 11 |  | 7 |  |
| BPPCT017 | 10 | 5 | 10 |  | 10 | 199 (0.012)^c^, 207 (0.015)^c^ | 5 |  |
| BPPCT038 | 13 | 9 | 13 | 149 (0.018)^c^ | 11 | 131 (0.012)^c^, 133 (0.147)^c^, 135 (0.006)^c^, 143 (0.047)^c^ | 11 |  |
| AMPA100 | 8 | 7 | 8 | 218 (0.059)^c^ | 8 | 210 (0.024)^c^ | 7 |  |
| BPPCT008 | 9 | 6 | 8 | 107 (0.081)^c^ | 9 | 125 (0.056)^c^ | 7 |  |
| BPPCT025 | 9 | 5 | 9 | 153 (0.035)^c^ | 9 |  | 5 |  |
| CPPCT030 | 15 | 8 | 13 |  | 15 | 169 (0.018)^c^, 173 (0.015)^c^ | 10 |  |
| Ma014a | 6 | 3 | 6 |  | 6 | 136 (0.009)^c^ | 3 |  |
| Ma040a | 11 | 6 | 9 | 211 (0.026)^c^, 227 (0.098)^c^ | 9 | 213 (0.024)^c^ | 10 |  |
| UDP98-412 | 11 | 6 | 10 | 111 (0.109)^c^, 113 (0.009)^c^ | 11 | 93 (0.018)^c^ | 7 |  |
| CPPCT022 | 12 | 9 | 11 | 246 (0.026)^c^, 250 (0.028)^c^, 262 (0.012)^c^, 274 (0.024)^c^ | 12 |  | 10 |  |
| CPPCT033 | 9 | 6 | 9 | 139 (0.040)^c^ | 9 | 141 (0.018)^c^, 147 (0.044)^c^, 161 (0.035)^c^ | 6 |  |
| CPPCT006 | 10 | 7 | 10 | 193 (0.090)^c^, 195 (0.038)^c^ | 10 | 175 (0.077)^c^ | 7 |  |
| UDP98-409 | 17 | 12 | 16 | 130 (0.006)^c^, 132 (0.125)^c^, 156 (0.012)^c^ | 16 | 122 (0.062)^c^, 140 (0.093)^c^, 150 (0.009)^c^ | 14 | 154 (0.092)^c^ |
|  | 239 | 143 | 224 | 23 (0.089)^d^ | 232 | 32 (0.124)^d^ | 162 | 1 (0.003)^d^ |

^1^ total allele observed at each locus

^2^alleles shared by at least two of the three clusters

^a^ alleles observed in each cluster pairwise (clusters 1 *vs.* 3, 1 *vs.* 4 and 3 *vs.* 4)

^b^ specific alleles observed in each cluster pairwise (clusters 1 *vs.* 3, 1 *vs.* 4 and 3 *vs.* 4)

^c^ frequency of alleles based on the total number of alleles observed at the 25 SSR loci in the 207 apricot accessions studied

^d^ frequency of specific alleles based on the total number of alleles detected at the 25 loci
